# Supplementary material for: Resolving the full spectrum of human genome variation using Linked-Reads
Source: Genome Res. 2019 Apr;29(4):635–45. doi: 10.1101/gr.234443.118 (PMC6442396; doi:10.1101/gr.234443.118)
Supplement: Supplemental Material [file supp_gr.234443.118_Supplemental_longranger-2.2.zip › longranger-2.2.tar.bz2!/lr2.2-public/lib/stan/cmdstan/stan/doc/api/html/trace_8hpp_source.html]

Stan: src/stan/math/matrix/trace.hpp Source File


|  |  |
| --- | --- |
|  | Stan  2.5.0  probability, sampling & optimization |


- Main Page
- Related Pages
- Namespaces
- Classes
- Files

- File List
- File Members

All Classes Namespaces Files Functions Variables Typedefs Enumerations Enumerator Friends Macros Pages

- src
- stan
- math
- matrix

trace.hpp

Go to the documentation of this file.

1 #ifndef STAN\_\_MATH\_\_MATRIX\_\_TRACE\_HPP

2 #define STAN\_\_MATH\_\_MATRIX\_\_TRACE\_HPP

3

4 #include <stan/math/matrix/Eigen.hpp>

5 #include <stan/meta/traits.hpp>

6

7 namespace stan {

8  namespace math {

9

19  template <typename T>

20  inline T trace(const Eigen::Matrix<T,Eigen::Dynamic,Eigen::Dynamic>& m) {

21  return m.trace();

22  }

23

24  template <typename T>

25  inline T

26  trace(const T& m) {

27  return m;

28  }

29  }

30 }

31 #endif

traits.hpp

Eigen.hpp

stan::math::trace

T trace(const Eigen::Matrix< T, Eigen::Dynamic, Eigen::Dynamic > &m)

Returns the trace of the specified matrix.

**Definition:** trace.hpp:20


---

[ Stan Home Page ]

*© 2011–2014,
Stan Development Team.*
